# Effectiveness of Self-Monitoring Approach Using Fitness Trackers to Improve Walking Ability in Rehabilitation Settings: A Systematic Review

**DOI:** 10.3389/fresc.2021.752727

**Published:** 2021-12-02

**Authors:** Eri Otaka, Kazuyo Oguchi, Izumi Kondo, Yohei Otaka

**Affiliations:** ^1^Assistive Robot Center, National Center for Geriatrics and Gerontology, Obu, Japan; ^2^Department of Rehabilitation Medicine, Kariya Toyota General Hospital, Kariya, Japan; ^3^Department of Rehabilitation Medicine I, School of Medicine, Fujita Health University, Toyoake, Japan

**Keywords:** activity monitors, motivation, pedometers, rehabilitation, self-monitoring, wearable devices, behavioral change, walking ability

## Abstract

**Background:** A self-monitoring approach utilizing fitness trackers that provide feedback regarding physical activities has been recently applied to rehabilitation patients to promote voluntary walking activities. Although this approach has been proven to increase physical activity, it is uncertain whether the intervention improves walking ability.

**Aim:** This review investigated whether the additional self-monitoring approach using activity trackers would improve walking ability in any type of rehabilitation setting.

**Methods:** A systematic search was performed in four databases [PubMed (MEDLINE), The Cochrane Library, SPORTDiscus, and Cumulative Index to Nursing and Allied Health Literature] to identify studies that examined the self-monitoring approach combined with rehabilitative intervention vs. the same rehabilitative intervention only in participants with any unhealthy conditions. Two review authors independently assessed the eligibility of all the retrieved English literature published from 2009 to 2019, then discussed the final inclusion. The risk of bias was assessed referring to the criteria of the Cochrane Risk of Bias tool. The key findings were synthesized using narrative synthesis. In addition, a quantitative synthesis was conducted when more than two studies investigating the same disease were identified.

**Results:** Eleven randomized controlled trials satisfied the eligibility criteria, nine of which had a lower risk of bias. The types of diseases included stroke, chronic obstructive pulmonary disease (COPD), cancer, Parkinson's disease, hemophilia, peripheral artery disease, post-total knee arthroplasty, and geriatric rehabilitation. Eight studies reported measures of walking endurance and four reported measures of gait speed. In the quantitative synthesis of two studies investigating COPD, there was a significant between-group difference in terms of changes in the 6-min walking distance from the baseline, which was favorable to the additional self-monitoring intervention group (mean difference: 13.1 m; 95% confidence interval, 1.8–24.5; 2 studies, 124 participants; *p* = 0.02; *I*^2^ = 0%). Other available data revealed no consistent evidence regarding effectiveness of the intervention.

**Conclusions:** The findings indicate that there is little evidence suggesting the effectiveness of the self-monitoring approach in improving walking ability in rehabilitation settings. However, a weak recommendation for patients with stable COPD was implicated in the quantitative synthesis. Further research would be required to explore the best indications for this self-monitoring approach.

**Systematic Review Registration:** CRD 42020157695.

## Introduction

According to research in motor skill learning, an increase in the amount of training leads to greater efficacy (1). When this theory is applied in rehabilitation medicine for achieving better walking ability, it is essential to increase the dose of walking activity (2, 3). However, in clinical practice, the number of steps taken per day by those living with disabilities are extremely low (4, 5), and only training sessions provided by physiotherapists are often inadequate to increase the real-world walking activity in daily life (6, 7). Therefore, effective techniques are required to motivate behavioral change in various rehabilitation settings (8).

Recently, a self-monitoring approach using wearable fitness trackers or activity monitors (pedometers or accelerometers) has been applied to promote voluntary walking activity as a part of rehabilitative intervention. This strategy aims to encourage patients to increase their physical activity by displaying their current activity/inactivity and providing specific information for subsequent improvement (9). Additionally, this kind of approach is getting more inexpensive and practical as many types of high-quality consumer-based pedometers have been made available recently (9). In cardiac rehabilitation, some studies involving the use of pedometers reported significant increase in the physical activity in patients after discharge or with low motivation (10, 11). Several systematic reviews have also revealed significant increase in the physical activity of some populations such as chronic obstructive pulmonary disease (COPD), cardiovascular diseases, and people with sedentary habits (12–15). Regarding stroke, a Cochrane review (16) confirmed that some studies reported the effectiveness of activity monitoring in increasing physical activity. However, these previous reviews raise the following questions. First, it remains unclear whether the increase in physical activity gained by self-monitoring is sufficient for improving walking ability, which is the ultimate goal of gait rehabilitation. Second, many of the included studies provided not just self-monitoring but a comprehensive program as intervention or did not provide any rehabilitative approach for the control groups, if any. Consequently, the pure effectiveness of a self-monitoring approach alone has yet to be proved when added to the usual rehabilitation.

Therefore, this review summarized the available evidence regarding the effectiveness of the self-monitoring approach using fitness trackers for actually improving walking ability, not merely increasing physical activity, in rehabilitation settings. Particularly, we reviewed the studies that compared the effects of the self-monitoring approach in addition to rehabilitation with the same rehabilitation alone.

## Materials and Methods

The study adheres to the Preferred Reporting Items for Systematic Reviews and Meta-Analyses (PRISMA) statement (17, 18) and the checklist. The protocol was registered with the International Prospective Register of Systematic Reviews (CRD 42020157695).

### Review Question

The research question was as follows: Does an additional self-monitoring approach using wearable activity trackers result in improved walking ability compared to rehabilitative intervention alone?

### Data Sources

A systematic search of the literature was performed using four different electronic databases [PubMed (MEDLINE), The Cochrane Library, SPORTDiscus, and Cumulative Index to Nursing and Allied Health Literature (CINAHL)]. We developed individualized search strategies for each database (Appendix A).

### Study Selection

#### Types of Study Design

Randomized controlled trials (RCTs) and non-RCTs were included.

#### Types of Participants

We included studies with participants who underwent rehabilitative intervention due to older age, frailty, various disorders, or any unhealthy conditions, regardless of the disease type. Studies regarding children, adolescents, or healthy adults (<65 years of age with no need for rehabilitative intervention) were excluded.

#### Types of Intervention

We included all trials that examined the self-monitoring approach combined with rehabilitative intervention vs. the same rehabilitative intervention only. The self-monitoring approach is defined as a method designed to promote physical activity by wearing any type of pedometer, activity monitor, fitness tracker, or accelerometer. These devices were intended to encourage participants to be more active by recording and displaying their daily activities—counting the number of steps walked daily, recording the time spent in moderate-to-vigorous intensity physical activity, or calculating the calories expended. Based on these records, participants receive various types of feedback, including daily records, weekly summaries, or sometimes individualized tasks, instructions, or goals based on a specific protocol. Therefore, the devices adopted in this intervention had to possess the capability to retain data for the preceding several days in stored memory, thereby enabling the average or total count for a certain period to be evaluated. Some of these devices have functions to display cumulative step counts for each day so that the user could see the step counts immediately and daily, but this type of function was not considered necessary for providing regular feedback.

#### Types of Comparators

We included studies in which participants in the control group had the same health conditions as the intervention group and the same rehabilitative intervention was provided for both groups, except for the self-monitoring approach.

#### Types of Outcome Measures

We defined the primary outcome domain of interest as the quantified walking ability measured by the gait speed or walking endurance (timed walking distance) at the end of the intervention. Moreover, we adopted any physical functions related to walking ability by measuring the balance (Berg Balance Scale, Timed Up and Go Test), aerobic capacity (maximum oxygen uptake), muscle strength or power (handgrip strength, quantified knee extension muscle strength, and 30-s chair stand test) at the end of the intervention as additional outcomes of interest. It was considered eligible if the outcome analysis included any of the final values or changes from baseline. Whether the outcomes of interest were the primary outcomes of the study was not considered.

#### Study Selection Procedure

After eliminating irrelevant studies by examining the titles and abstracts, the full texts of all remaining studies were obtained, and two independent review authors (EO and YO) assessed the eligibility of all the manuscripts. After disagreements between individual judgments were discussed, consensus was gained and the decision of the studies to be ultimately included was finalized.

### Data Extraction

Two independent review authors (EO and YO) performed the data extraction. Study investigators were contacted for unreported data if necessary to complete the description of the included studies.

### Assessing Risk of Bias

The risk of bias was assessed by collaboration of the two review authors (EO and YO), and disagreements were resolved by discussion. The criteria of the Cochrane risk of bias tool (19) were used to evaluate the methodological quality. We also inspected funnel plots to assess the risk of publication bias.

### Data Synthesis

At the registry, we did not intend to conduct a meta-analysis because we did not anticipate that there would be ≥2 studies with similar participant populations, interventions, and outcome measures of interest. However, we identified a small number of similar studies that evaluated the effect of the self-monitoring approach for the same disease; we therefore partially conducted a quantitative synthesis by pooling the data using Review Manager 5.3 (Nordic Cochrane Centre, The Cochrane Collaboration, 2014). We undertook both a fixed-effect model and a random-effects model in synthesizing the studies and obtained the same results in all cases. In addition, we used the mean difference and 95% confidence interval (CI) to summarize studies using the same measurement scales in the analyses, and standardized mean difference and 95% CIs when the studies adopted different scales to assess the same outcome. *I*^2^ statistics were used to assess the heterogeneity. Regarding the remaining included studies, we provided a narrative synthesis as planned a priori, giving priority to studies with a lower risk of bias.

## Results

### Flow Diagram of the Studies Retrieved for Review

**Figure 1:**
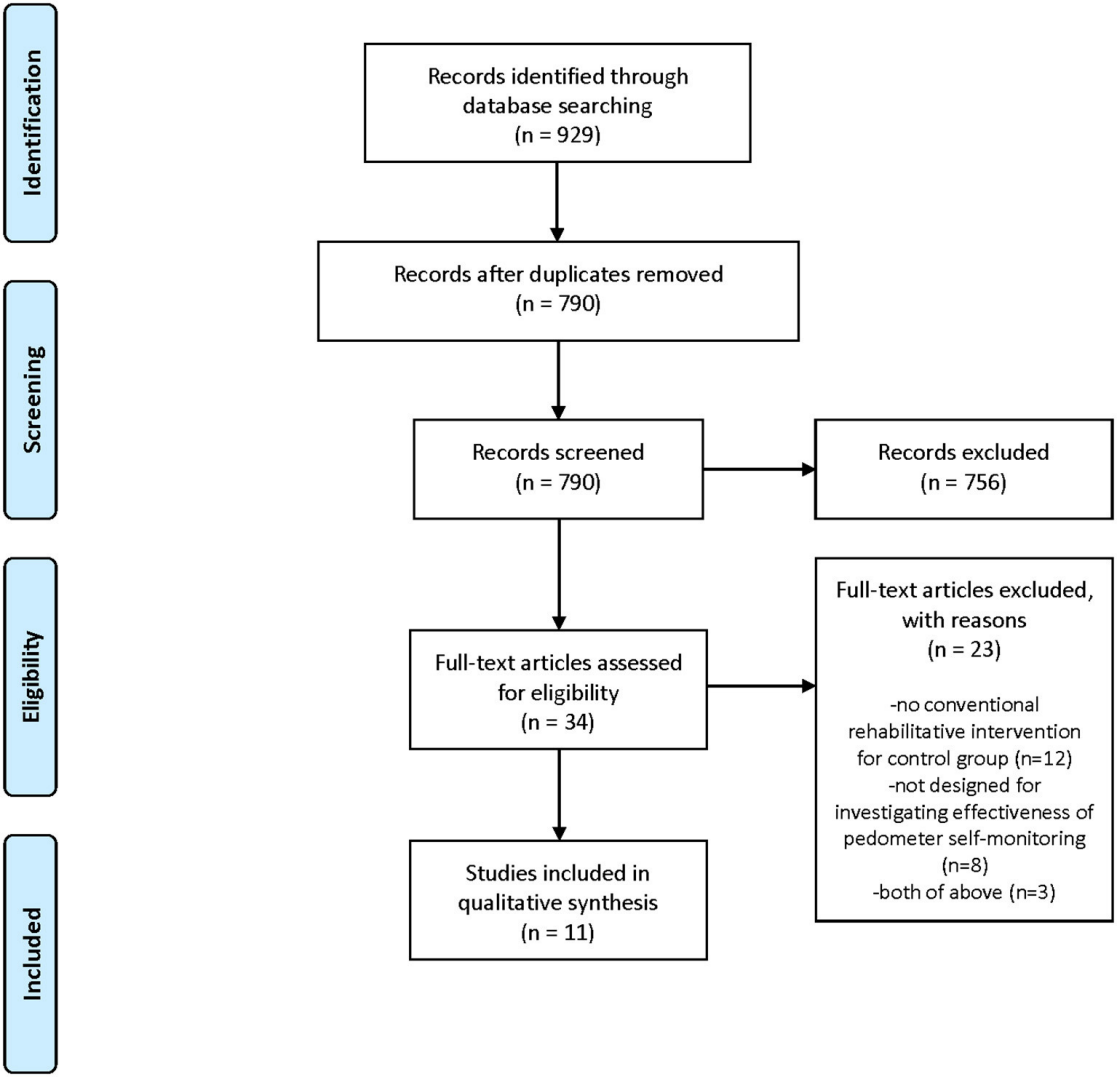
PRISMA flow diagram of the literature search and study selection.

Figure 1 depicts a flow diagram of the study selection for this review. We identified 929 references from our search. Of these, we removed 139 duplications, and eliminated 756 by screening the titles and abstracts. From the 34 remaining articles, we excluded 23 and finally included 11 studies in the review. Appendix B provides the excluded studies at the full-text stage and the reasons for exclusion. Exclusion from the final selection was because no rehabilitative intervention was provided for the control group (15 studies), or because the study was not designed to investigate the effectiveness of a solely self-monitoring approach, that is, the investigated intervention was a comprehensive program including a self-monitoring approach as well as dietary or psychological approaches that were not provided for the control group (10 studies).

### Characteristics of Included Studies

A detailed description of the 11 studies included is presented in Tables 1, 2. The study participants in these studies had a wide variety of diseases, including stroke (20, 21), COPD (22, 23), cancer (24, 25), Parkinson's disease (26), hemophilia (27), peripheral artery disease (29), post-total knee arthroplasty (30), and geriatric rehabilitation in various diseases (28). Regarding study settings, two studies (21, 28) recruited inpatients during rehabilitation and others targeted outpatients. Consequently, the duration of study intervention varied from within 1 month (21, 28) to 24 months (26).

**Table 1:** Characteristics of the included studies 1: Study design and methods.

**References/ country**	**Study design/sample size/number of arms**	**Participants**		**Intervention**				**Control**			
		**Inclusion criteria**	**Setting/ recruitment**	**Program**	**Frequency**	**Duration**	**Type of device**	**Program**	**Frequency**	**Duration**	**Device use**
Danks et al. (20) USA	RCT *N* = 37 2 arms	Stroke >6 months, <10,000 steps per day	Outpatients of local clinics, support groups, newspaper advertisements	(*n* = 19) fast-walking training (50 min) + self monitoring -advice from therapists -goals based on the averaged steps per day	3 per week	12 weeks	StepWatch Activity Monitor	(*n* = 18) fast-walking training (50 min)	3 per week	12 weeks	No
Dorsch et al. (21) International	RCT *N* = 37 2 arms	Stroke within 35 days	Inpatients of rehabilitation centers	(*n* = 73) inpatient rehabilitation + self-monitoring -advice from therapists	3 per week	Median 22.5 days (from enroll to discharge)	GulfCoast Data Concepts	(*n* = 78) inpatient rehabilitation + feedback of gait speed	3 per week	Median 20 days (from enroll to discharge)	Yes
Kawagoshi et al. (22) Japan	RCT *N* = 37 2 arms	COPD stable for 3 months	Patients at home	(*n* = 19) multidisciplinary home-based program + self-monitoring -advice from therapists -goal of 8,000 steps per day	Every 2 weeks (exercise supervision) + 1 per month (advice)	1 year	Kens Lifecorder EX	(*n* = 20) multidisciplinary home-based program	Every 2 weeks	1 year	No
Mendoza et al. (23) Chile	RCT *N* = 37 2 arms	COPD stable for 4 weeks	Outpatients of public primary health centers	(*n*= 52) counseling (30 min) + self-monitoring -advice from therapists -goals based on the averaged steps per day	1 per month	3 months	PD724 Tanita	(*n* = 50) counseling (30 min)	1 per month	3 months	No
Uhm et al. (24) Korea	RCT *N* = 37 2 arms	Breast cancer post primary treatment	Three hospitals (details unknown)	(*n* = 179) home-based exercise + self-monitoring -via smartphone application -goals based on the initial physical function	1 per week	12 weeks	InBodyBand	(*n* = 177) home-based exercise	Details unknown	12 weeks	No
Mayo et al. (25) Canada	RCT *N* = 37 3 arms	Cancer (various, advanced) fatigue 4–10 by visual analog scale	Outpatients of a University health center	(intervention 1: *n* = 8, intervention 2: *n* = 10) multidisciplinary home-based program + self-monitoring -advice by phone call -goals based on the averaged steps per day -intervention 1: DURING the program -intervention 2: AFTER the program	1 per week	8 weeks	(details unknown)	(*n* = 8) multidisciplinary home-based program	Details unknown	8 weeks	No
Van Nimwegen et al. (26) Netherlands	RCT *N* = 540 2 arms	Parkinson's disease Hoehn and Yahr stage ≤ 3	32 community hospitals	(*n* = 299) physiotherapy sessions (35/year) + self-monitoring -advice from therapists -systematic goal setting	1 per month	24 months	Triaxial accelerometer (details unknown)	(*n* = 287) physiotherapy sessions (35/year)	35/year	24 months	Yes (displays concealed)
Goto et al. (27) Japan	RCT *N* = 37 2 arms	Hemophilia	Outpatients of 4 hospitals	(*n* = 19) home-based exercise program + self-monitoring -monitored using Internet	(details unknown)	8 weeks	HJA-350IT Omron	(*n* = 18) home-based exercise program (resistance training)	(details unknown)	8 weeks	Yes (displays concealed)
Peel et al. (28) Australia	RCT *N* = 270 2 arms	Geriatric rehabilitation patients	Inpatients of post-acute care geriatric rehabilitation units at 3 sites	(*n* = 135) inpatient rehabilitation (details unknown) + self-monitoring -advice from therapists	1 per week	median 15 days (4 weeks or until discharge)	ActivPal	(*n* = 135) inpatient rehabilitation (details unknown)	(details unknown)	median 14 days (4 weeks or until discharge)	Yes
Nicolaï et al. (29) Netherlands	RCT *N* = 304 3 arms	Peripheral artery diseases: Fontaine stage 2	Outpatient of 11 vascular surgery clinics	(*n* = 93) supervised exercise therapy by physical therapists (30 min) + self-monitoring -advice from therapists	(details unknown)	12 months	Personal Activity Monitor	(control 1: *n* = 102, control 2: *n* = 109) control 1: walking advice and brochure control 2:supervised exercise therapy by physical therapists	2–3 per week	12 months	No
Smith et al. (30) USA	RCT *N* = 60 2 arms	Post-total knee arthroplasty 10–18 months postoperatively	Outpatients of surgical follow-up clinics	(*n* = 30) home-based exercise program + self-monitoring (details unknown)	1 per week	16 weeks	Fitbit One, Fitbit Flex	(*n* = 30) home-based exercise program	1 per week	16 weeks	No

*RCT, randomized controlled trial; COPD, chronic obstructive pulmonary disease*.

**Table 2:** Characteristics of the included studies 2: Results and other information.

**References/ country**	**Attendance rate at rehabilitation programs**	**Time for device use**	**Amount of walking activity**	**Outcomes**			**Conflict of interests**	**Funding**
				**Outcomes of Interest**	**Mean difference at the end of intervention (IV, fixed, 95% CI)**	**Mean difference in change from baseline (IV, fixed, 95% CI)**		
Danks et al. (20) USA	*Intervention group:* 80.6% (29 ± 3/36 sessions) *Control group:* 75.0% (27 ± 2/36 sessions)	(Details unknown)	*Intervention group:* pre 4,146 ± 2,550 steps, 1.39 ± 0.62 h/day post 5,160 ± 2,504 steps^†^, 1.70 ± 0.79 h/day^†^ *Control group:* pre 6,080 ± 3,015 steps, 2.09 ± 0.88 h/day post 7,087 ± 3962 steps^†^, 2.35 ± 1.17 h/day^†^ (between-group difference: n.s.)	(a) 6-min walking distance (m) (b) Gait speed (self-selected, 10 m) (m/s) (c) Gait speed (max, 10 m) (m/s)	(a) 0.00 (−0.75 to 0.75) (b) −0.08 (−0.39 to 0.23) (c) −0.10 (−0.86 to 0.65)	(Not available)	none	National Institutes of Health
Dorsch et al. (21) Inter-national	(Details unknown)	*All participants:* sensor data obtained for 84.4% of all study days	*Intervention group:* 16.6 ± 14.3 min/day spent in walking *Control group:* 15.1 ± 13.1 min/day spent in walking (between-group difference: *p* = 0.54)	(a) 3-min walking distance (m) (b) Gait speed (max, 15 m) (m/s)	(a) −0.60 (−48.93 to 47.73) (b) −0.02 (−0.19 to 0.15)	(Not available)	None	NIH/NICHD NIH/NCATS
Kawagoshi et al. (22) Japan	*All participants:* 65.4 ± 6.8% (daily program at home for 239 ± 25 days of a year)	*Intervention group:* 80.4 ± 13.3% (293 ± 49 days of a year)	*Intervention group:* 51.3 ± 63.7 min/day spent in walking *Control group:* 12.3 ± 25.5 min/day spent in walking (between-group difference: *p* = 0.04)	(a) 6-min walking distance (m) (b) Knee muscle strength (kg)	(a) −22.00 (−131.25 to 87.25) (b) 3.50 (−6.41 to 13.41)	(a) 13.20 (−26.13 to 52.53) (b) 2.82 (−0.11 to 5.75)	None	(Not stated)
Mendoza 2014 Chile	(Details unknown)	*All participants:* all stated that they had used the pedometer as instructed	*Intervention group:* 3,080 ± 3254.8 steps /day increased *Control group:* 138.3 ± 1,950.4 steps/day increased (between-group difference: *p* <0.001)	6-min walking distance (m)	4.40 (−23.53 to 32.33)	13.10 (1.24, 24.96)	None	Fondo Nacional de Investigacion Desarrollo en Salud
Uhm et al. (24) Korea	(Details unknown)	(Details unknown)	(Details unknown)	(a) 2-min walking distance (m) (b) 30-s chair stand (times) (c) Handgrip strength (kg)	(a) 3.60 (−4.33 to 11.53) (b) 0.10 (−1.49 to 1.69) (c) 0.30 (−0.57 to 1.17)	(Not available)	None	National Information Society Agency funded by the Ministry of Science, ICT and Future Planning
Mayo et al. (25) Canada	(Details unknown)	*Intervention group:* 8 of 18 (44.4%) participants had sufficient pedometer data	*Intervention group:* mean 223 (range 9–514) steps /day increased	2-min walking distance (m)	(Not available)	(Not available)	None	MUHC and Research Institute of Pilot Project Competition
Van Nimwegen et al. (26) Netherlands	*Intervention group:* 38.9% (mean 13.6/35 sessions) *Control group:* 37.1% (mean 13.0/35 sessions)	(Details unknown)	*Intervention group:* 38.7 kcal/day increased *Control group:* −14.2 kcal/day increased (between-group difference: *p* <0.001)	6-min walking distance (m)	9.40 (−7.84 to 26.64)	13.31 (−3.21 to 29.83)	Non	Netherlands Organization for Health Research and Development
Goto et al. (27) Japan	*Intervention group:* 79.0 ± 16.6% *Control group:* 32.8 ± 21.1% (days in 8 weeks)	*Intervention group:* 90.3% (range 62.5–100) (days in 8 weeks)	*Intervention group:* 5,805.6 ± 3,384.0 steps /day, 86.0 ± 45.6 min/day post intervention *Control group:* 4,910.2 ± 2,663.5 steps /day, 75.0 ± 34.9 min/day post Intervention (between-group difference: n.s.)	(a) Timed 10-m walk (s) (b) Knee muscle strength (kg) (c) Modified functional reach (cm)	(a) −0.10 (−0.87 to 0.67) (b) 0.07 (−0.42 to 0.56) (b) −0.10 (−5.46 to 5.26)	(Not available)	None	Grants from the Tokyo Physical Therapy Association and the Japan Society for the Promotion of Rehabilitation
Peel et al. (28) Australia	(Details unknown)	*All participants:* worn continuously or with no difference between groups	*Intervention group:* mean 24.6 min/day of non-therapy walking *Control group:* mean 17.3 min/day of non-therapy walking (between-group difference: *p =* 0.001)	Gait speed (self-selected, 3–4 m) (m/s)	0.01 (−0.05 to 0.07)	0.04 (−0.02, 0.10)	None	Australian National Health and Medical Research Council (NHMRC) Grant
Nicolaï et al. (29) Netherlands	(Details unknown)	*Intervention group:* 22 patients (28.9%) reported NOT having used the device as instructed	(Details unknown)	(a) Absolute claudication distance (m) (b) Functional claudication distance (m)	(Not available)	(Not available)	None	The Netherlands Organization for Health Research and Development (ZonMw)
Smith et al. (30) USA	*Intervention group:* 78.6 ± 8.5% *Control group:* 74.8 ± 11.0%	(Details unknown)	(Details unknown)	(a) 6-min walking distance (m) (b) Knee muscle strength (kg)	(Not available)	(Not available)	None	FedEx Institute of Technology at the University of Memphis

*IV, inverse variance method; CI, confidence interval. (†) a significant change from pre- to post-intervention*.

The self-monitoring approach in the present review utilized various methods to show the monitored activity data and provide feedback to encourage increased activity. Most of the included studies provided data of activities directly from the physiotherapists during face-to-face appointments (20–23, 26, 28, 29) or phone calls (25), whereas two studies (24, 27) provided information using an Internet application, not accompanying face-to-face sessions with therapists. In addition, some studies had specific goals to be achieved, such as step goals based on the average steps per day (23), 8,000-step goal (22), weekly goal and achievement rate provided automatically (24), and systematic goal setting in physiotherapy sessions (26, 28).

For demonstrating the extent to which the interventions were delivered and practiced as intended, attendance rate for the rehabilitation program, time for device use, and amount of walking activity is shown in Table 2. These aspects are known to be important in the implementation of the study in real-world settings (31, 32). Attendance rate for rehabilitation program was reported in five studies (20, 22, 26, 27, 30) and varied from 30 to 80%. Regarding time for device use, only two studies reported precise data of the intervention groups (22, 27), in both of which participants wore pedometers for more than 80% of the days the intervention was held. One study (21) reported that the data of the accelerometers in all the participants (including both the intervention and the control groups) was obtained for more than 80% of the study days. Two studies (23, 28) simply described that the participants wore pedometers as intended, and two other studies (25, 29) stated that the participants had not used pedometers sufficiently. Regarding the amount of walking activity, seven studies (20–23, 26–28) reported that the walking activity increased more in the intervention group than in the control group.

Eight studies reported measurement of walking endurance (20, 22–26, 29, 30), most of which were 6-min walking distances (20, 22, 23, 30), and four reported measurement of gait speed (20, 21, 27, 28). For the secondary outcomes of interest, three studies (22, 27, 30) reported knee extension muscle strength. Handgrip strength (24), 30-s chair stand test (24) as a measure of muscle power, and Modified Functional Reach Test (27) as a measure of balance function were each found in single studies.

### Risk of Bias Assessment

**Figure 2:**
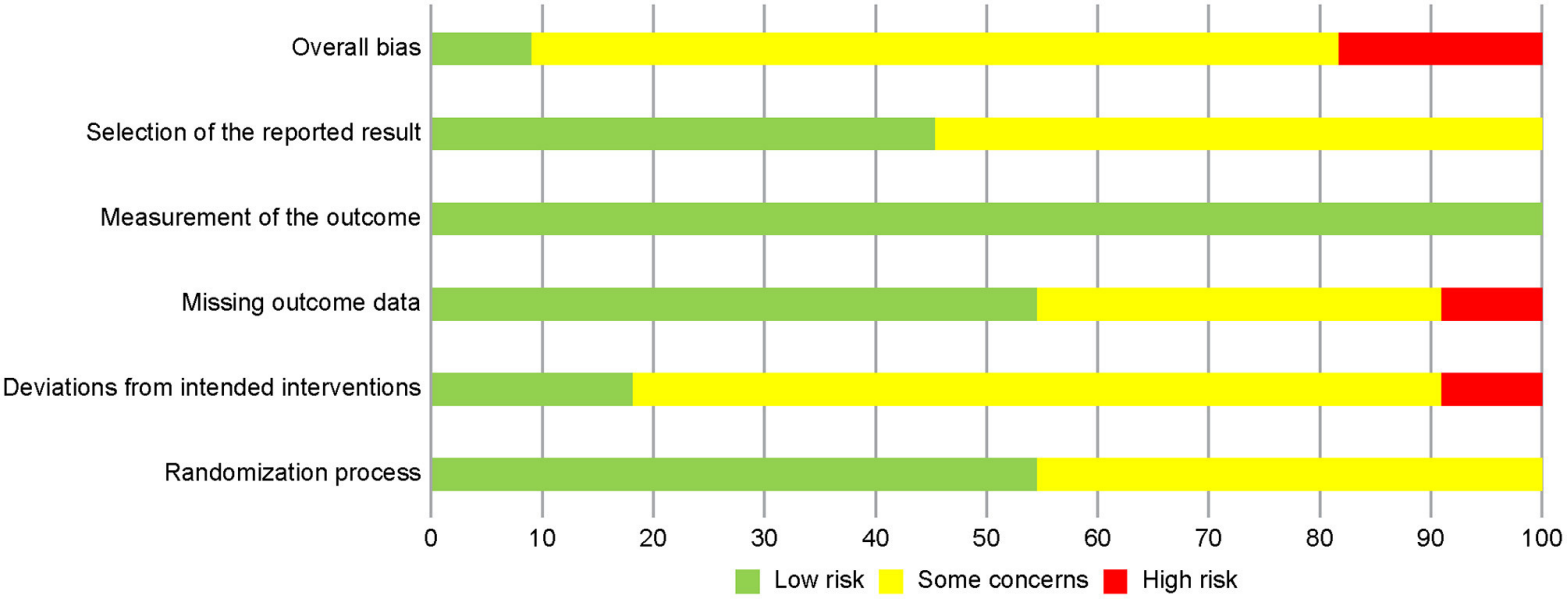
Risk of bias graph: review authors' judgments about each risk of bias item presented as percentages across all included studies.

Figure 2 summarizes the trials regarding risk of bias, which consists of five domains. The most prevalent risk of bias was in the area of Domain 2, deviations from the intended interventions, in which only 2 out of 11 studies were judged as having low risk. All the included studies failed to blind participants and therapists because the objective of the intervention was to make them aware of the participants' activity through use of the devices. The two studies with a low risk of bias reported information suggesting that deviations from the intended intervention that arose because of the trial context were unlikely to occur. In addition, six studies were judged as having some concerns in Domain 5, selection of the reported result, because we were unable to find protocols registered or published in advance.

**Figure 3:**
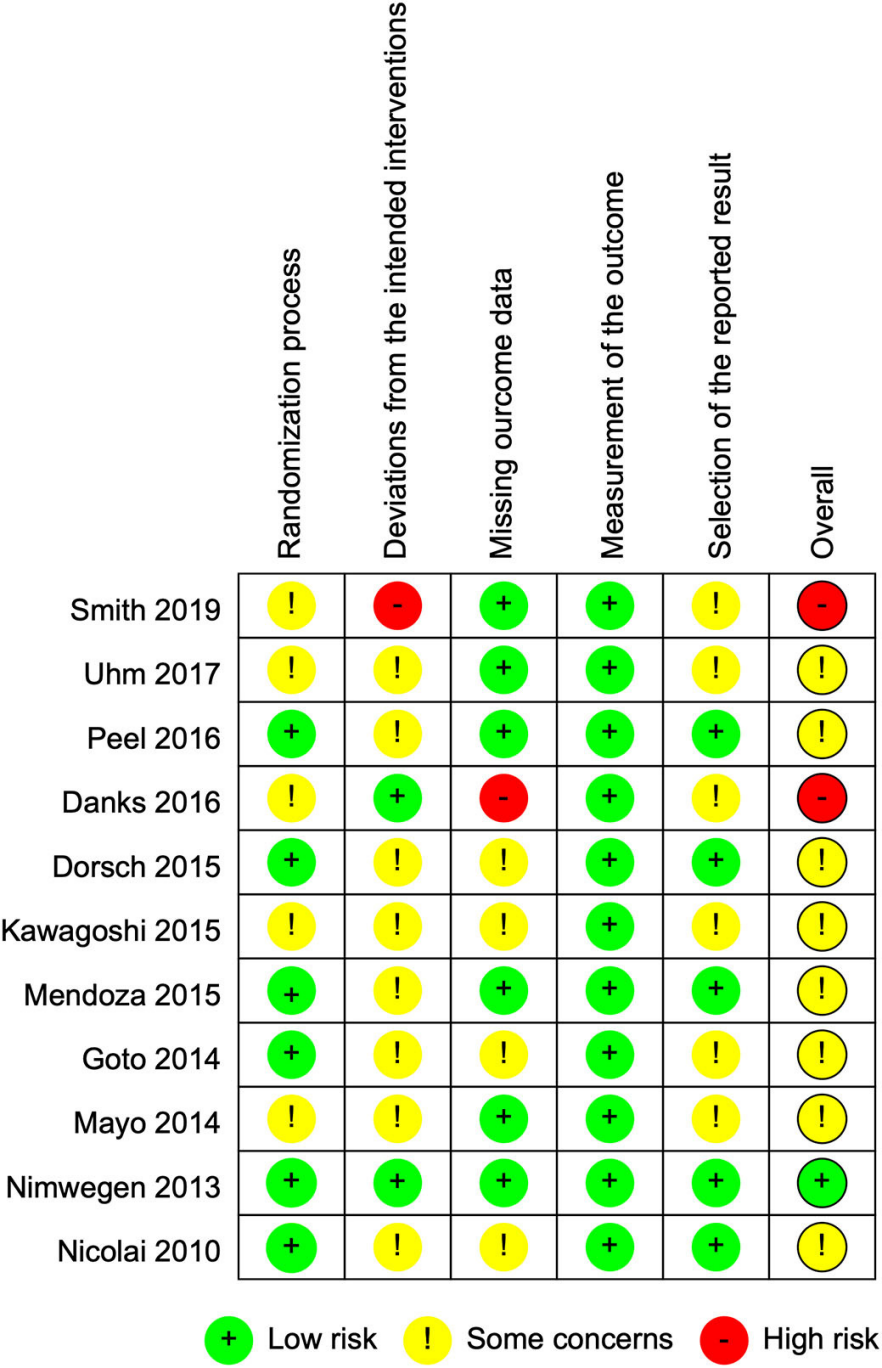
Risk of bias summary: review authors' judgments about each risk of bias item for each included study.

Figure 3 shows the risk of bias in each of the included studies. Only one study was judged as having a low risk of bias, and eight studies were judged to have some concerns. One study had a high risk in Domain 3, missing outcome data, because exacerbated pain caused dropouts in the intervention group only (20). Another study described no information regarding blindness of assessors and was therefore considered to have a high risk in Domain 2, deviations from the intended interventions (30).

### Synthesis of Key Findings—Walking Ability (Primary Outcome of Interest)

The included studies were categorized according to the types of diseases investigated as follows:

#### Stroke

One international study (21) investigated 151 inpatients with subacute stroke 1–3 months from onset. During the inpatient rehabilitation period, the effect of the additional provision of walking activity recorded by wireless sensors compared to the walking speed feedback only, was investigated. The other study (20) recruited 37 chronic outpatients more than 6 months post-stroke to examine the effect of a step activity monitoring program in addition to fast-walking training, which was perceived as having a high risk of bias, as mentioned above.

**Figure 4:**
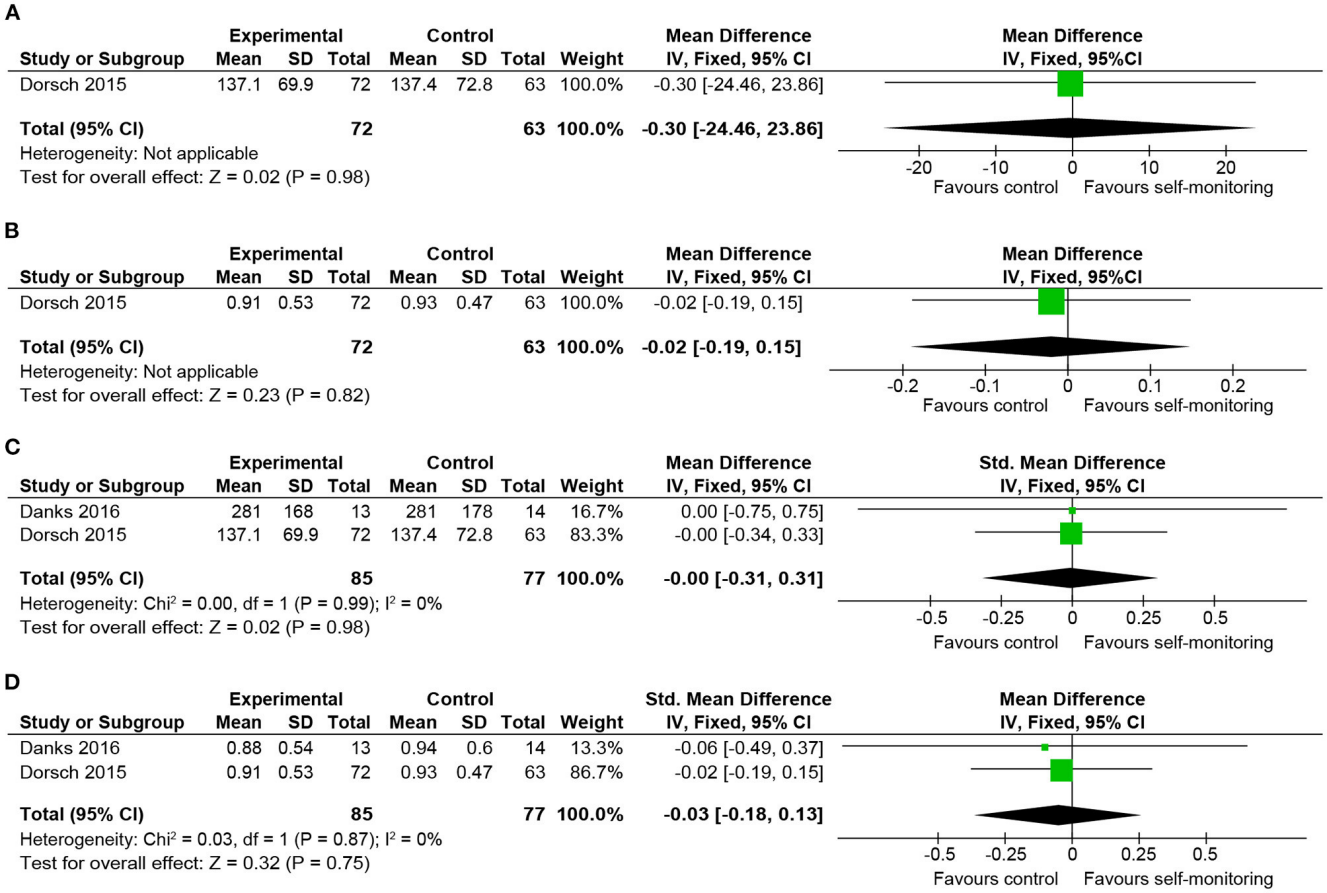
Comparison of walking ability in patients with stroke. **(A)** Walking endurance (3-min walking distance, m) post-intervention. **(B)** Gait speed (maximal, m/s) post-intervention. **(C)** Walking endurance (timed walking distance, m) post-intervention including a study at high risk of bias. **(D)** Gait speed (maximal, m/s) post-intervention including a study at high risk of bias. SD, standard deviation; IV, inverse variance method; CI, confidence interval.

Although time spent walking was longer in the intervention group, the post-intervention values in the study with a lower risk of bias revealed no between-group differences (Figures 4A,B). This trend was consistent with that observed when we conducted a quantitative synthesis combined with the study with a high risk of bias (6-min walking distance: standard mean difference, fixed-effect model: −0.00, 95% CI −0.31 to 0.31; 2 studies, 162 participants; *p* = 0.98; *I*^2^ = 0%, gait speed: mean difference, fixed-effect model: −0.03, 95% CI −0.18 to 0.13; 2 studies, 162 participants; *p* = 0.75; *I*^2^ = 0%) (Figures 4C,D).

#### COPD

Both included studies examined outpatients who were recently stable and evaluated the 6-min walking distance. One RCT (23) with 102 participants examined the effect of an additional self-monitoring approach with goals based on the averaged steps per day compared to monthly 30-min rehabilitative counseling with provision of a diary only. The intervention group showed significantly greater benefit upon changing the 6-min walking distance, although there was no significant between-group difference in the final value of the 6-min walking distance. Another RCT with 42 participants (22) provided additional feedback regarding physical activity with goal-setting of steps for outpatients undergoing a pulmonary rehabilitation program.

**Figure 5:**
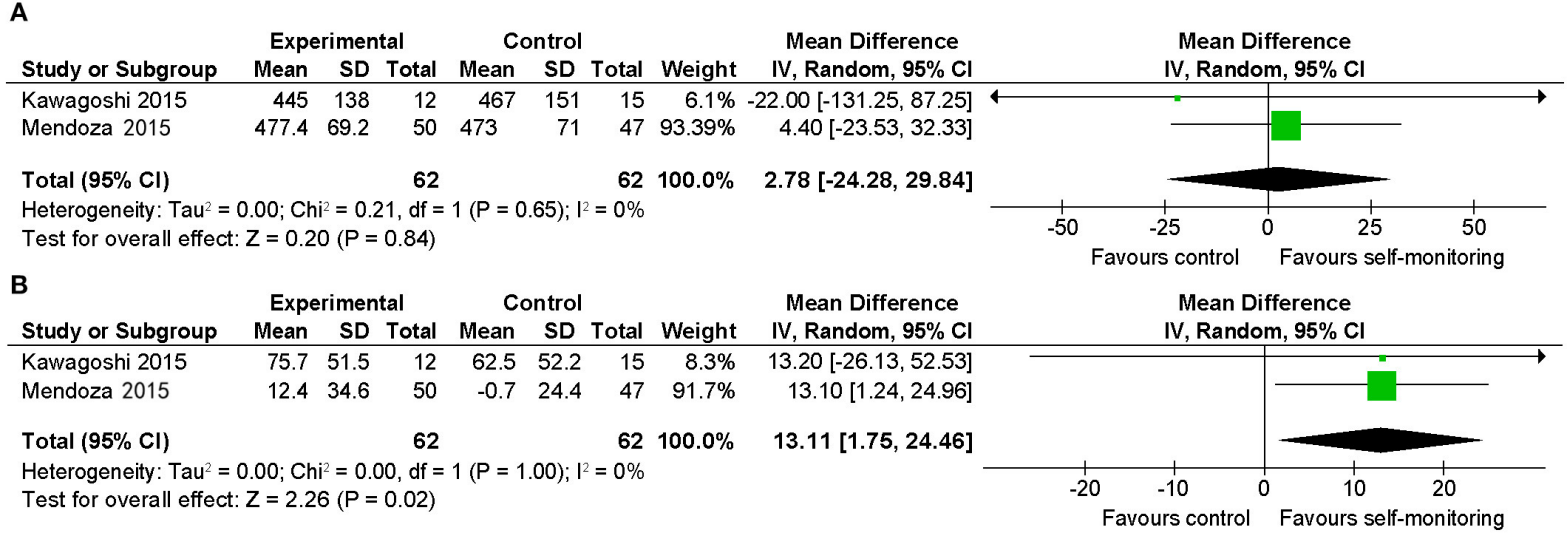
Comparison of walking ability in patients with stable COPD. **(A)** Walking endurance (6-min walking distance, m) post-intervention. **(B)** Walking endurance (6-min walking distance, m) change from baseline. SD, standard deviation; IV, inverse variance method; CI, confidence interval.

As a result of quantitative synthesis of the two studies, which showed significant increase in the amount of walking activity in the intervention groups, there was a between-group difference in the changes in 6-min walking distance, which was favorable to the intervention group and statistically significant (Figures 5A,B) (mean difference: 13.1 m; 95% CI, 1.8–24.4; 2 studies, 124 participants; *p* = 0.02; *I*^2^ = 0%). However, the mean difference in the changes from the baseline (13.1 m) was smaller than the minimal clinically important difference (MCID) reported in COPD (25–54 m) (33, 34).

#### Cancer

**Figure 6:**
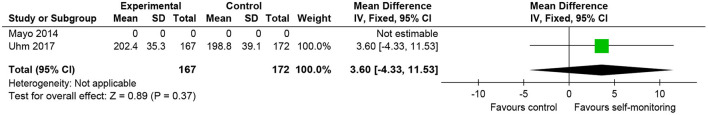
Comparison of walking ability in patients with cancer. Walking endurance (2-min walking distance, m) post-intervention. SD, standard deviation; IV, inverse variance method; CI, confidence interval.

One RCT (24) recruited 356 patients with breast cancer who completed primary cancer treatment. The post-intervention (12 weeks) value of 2-min walking distance was favorable to the intervention group (Figure 6). However, the difference was not statistically significant and the mean difference of post-intervention values (3.60 m) was smaller than the MCID reported in older adults (12.2–14.7 m) (35).

The other RCT (25) investigated two patterns of intervention for advanced cancer patients: self-monitoring during and after a home-based program, compared to a home-based program only. Although the outcomes included a 2-min walking distance, the data was not suitable for analyzing according to our protocol.

#### Parkinson's Disease

**Figure 7:**
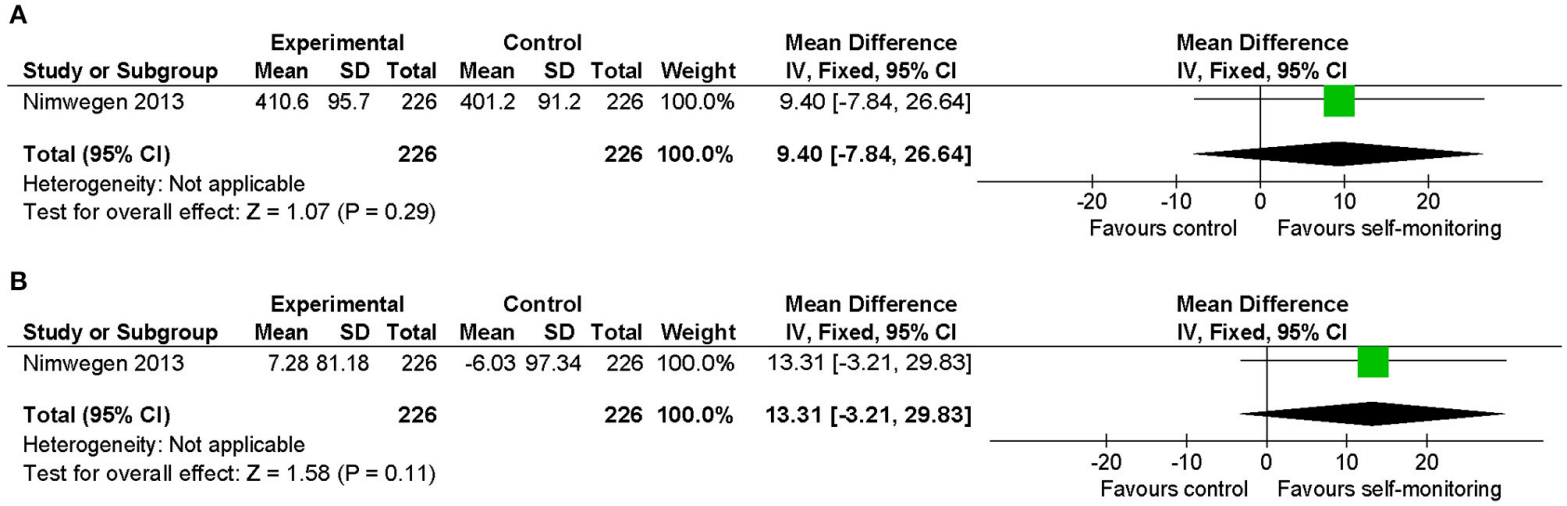
Comparison of walking ability in patients with Parkinson's disease. **(A)** Walking endurance (6-min walking distance, m) post-intervention. **(B)** Walking endurance (6-min walking distance, m) change from baseline. SD, standard deviation; IV, inverse variance method; CI, confidence interval.

The effect of additional self-monitoring with systematic goal setting compared to physiotherapy sessions only was examined in 540 sedentary patients with Parkinson's disease (26). Daily energy expenditure due to walking activity significantly increased in the intervention group. Both the post-intervention values and the change from baseline of the 6-min walking distance were favorable to the intervention group (Figures 7A,B). However, between-group differences in these indices were not statistically significant, and the mean differences of post-intervention values (9.40 m) and of changes from baseline (13.31 m) were smaller than the MCID reported in Parkinson's disease (82 m) (36).

#### Hemophilia

**Figure 8:**
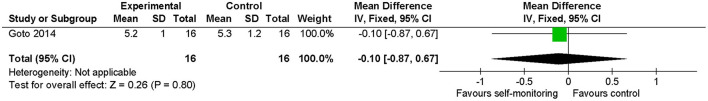
Comparison of walking ability in patients with hemophilia. Gait speed (timed 10-m walk, s) post-intervention. SD, standard deviation; IV, inverse variance method; CI, confidence interval.

One RCT investigated the effect of self-monitoring in addition to a home-based exercise program in 37 patients with hemophilia (27). The final value of the timed 10-m walk was favorable to the intervention group (Figure 8). However, the difference was not statistically significant and the mean difference (0.1-s) was very small when considered clinically.

#### Geriatric Rehabilitation

**Figure 9:**
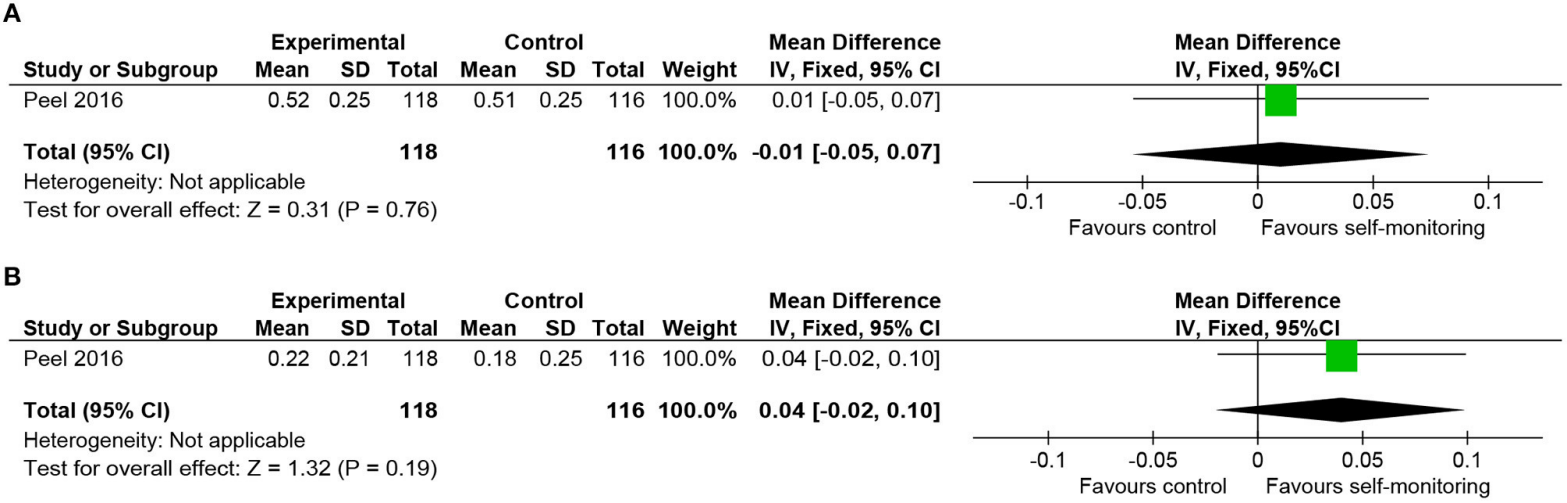
Comparison of walking ability in rehabilitation of geriatric patients. **(A)** Gait speed (self-selected, m/s) post-intervention. **(B)** Gait speed (self-selected, m/s) change from baseline. SD, standard deviation; IV, inverse variance method; CI, confidence interval.

We included one RCT in this category, which recruited 270 inpatients of post-acute geriatric rehabilitation units (28) and provided accelerometer data in addition to usual inpatient rehabilitation for the intervention group. The primary diagnoses included fractures, infections, neurological diseases, and cardiopulmonary diseases. Time spent walking was longer in the intervention group, and the change in gait speed from baseline was favorable to the intervention group. However, these differences were not statistically significant (Figures 9A,B). The mean difference of changes from baseline (0.04 m/s) was smaller than the MCID reported in heart failure (0.05 m/s) (37) or after hip fracture (0.10 m/s) (38).

#### Others

Two other studies that recruited patients with diseases in different categories [peripheral artery diseases (29) and post-total knee arthroplasty (30)] were included, but failed to obtain sufficient information to evaluate the effect of self-monitoring.

Finally, we inspected a funnel plot for the 6-min walking distance in patients with various diseases for reference. Publication bias was not graphically evident, although the number of included studies was small (Appendix C).

### Synthesis of Key Findings—Physical Functions Related to Walking Ability (Secondary Outcomes of Interest)

**Figure 10:**
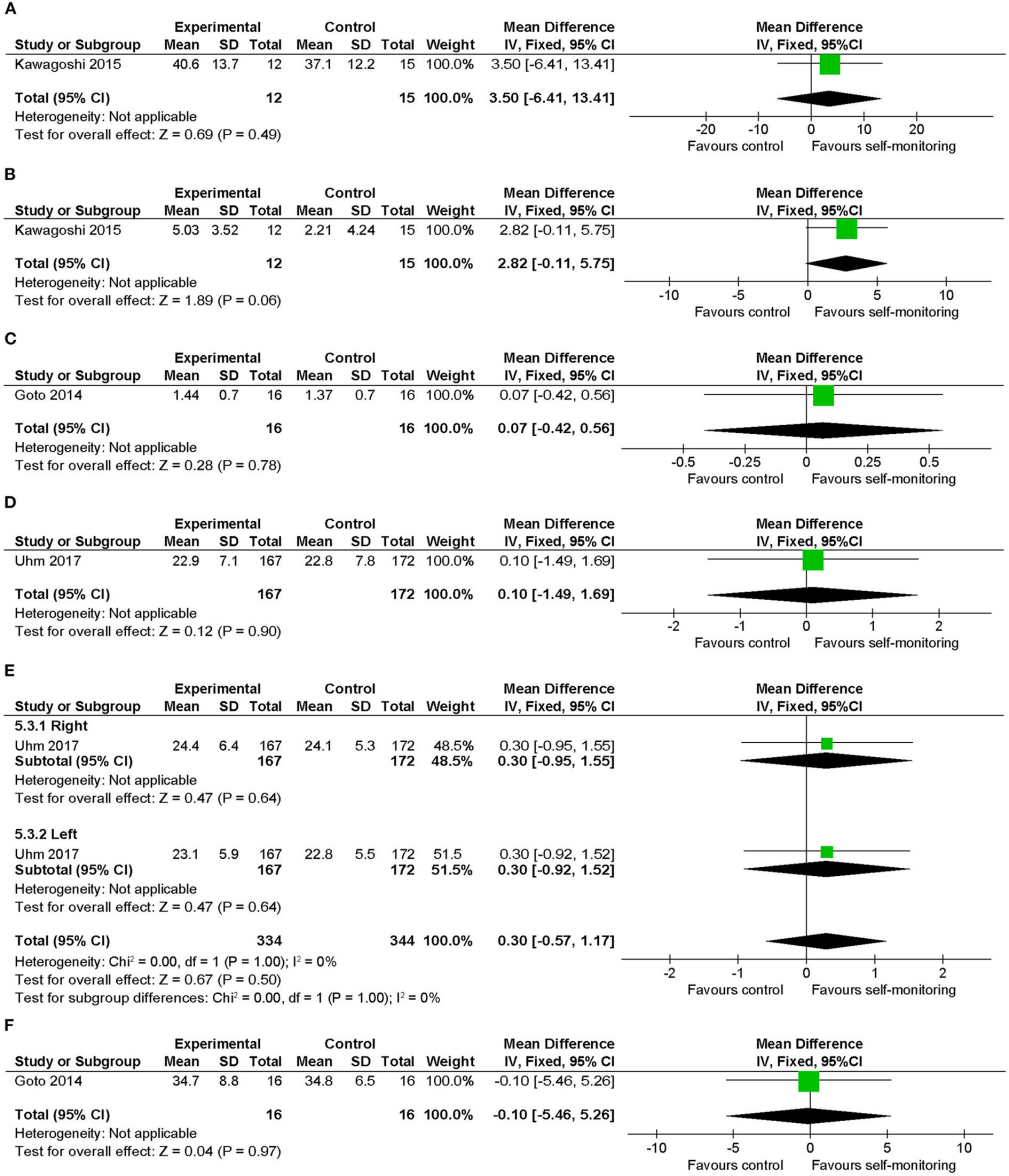
Comparison of physical functions related to walking ability (secondary outcomes of interest) (17). **(A)** Knee muscle strength (kg) post intervention in patients with stable COPD, **(B)** knee muscle strength (kg) change from baseline in patients with stable COPD, **(C)** knee muscle strength (kg) post intervention in patients with hemophilia, **(D)** 30-s chair-stand test (no.) post intervention in patients with cancer, **(E)** handgrip strength (kg) post intervention in cancer, **(F)** Modified Functional Reach Test (cm) post intervention in patients with hemophilia. SD, standard deviation; IV, inverse variance method; CI, confidence interval.

Regarding knee muscle strength, no significant difference was observed between the intervention and control groups in the data for stable COPD (Figures 10A,B) and hemophilia (Figure 10C). In addition, a chair-stand test (Figure 10D) and handgrip strength (Figure 10E) in patients with cancer and Modified Functional Reach Test (Figure 10F) in patients with hemophilia showed no between-group differences.

## Discussion

### Summary of Main Findings

This systematic review aimed to investigate the effectiveness of an additional self-monitoring approach using fitness trackers to improve walking ability when compared to rehabilitative intervention alone. We identified 11 studies that fulfilled the inclusion criteria and eight different types of diseases in these studies.

Among these, the most consistent evidence identified was a significant change from baseline in the 6-min walking distance in the self-monitoring group in patients with stable COPD. However, the mean effect of intervention did not achieve the MCID. In Parkinson's disease and post-acute geriatric rehabilitation conditions, the self-monitoring approach resulted in a significant increase in the amount of walking activity, and improvement in the walking ability was observed, but it was smaller than the MCID.

### Indication for the Self-Monitoring Approach

Overall, this review detected no specific type of disease with evident indications for self-monitoring, except for weak recommendations for patients with stable COPD. Some of the results in single RCTs favored the intervention, yet these changes were of little clinical importance, regardless of whether they were significant or not. Given these results, the effect of the self-monitoring approach might be relatively weak or non-existent, compared with the effect of rehabilitative intervention itself, which is customized for the targeted medical conditions.

Four studies (22, 23, 26, 28) reported increased physical activity with statistical significance and favorable results by self-monitoring, although three of the results were not statistically significant. The effectiveness of self-monitoring in increasing activity was consistent with previous studies (12–15). In addition, these results suggest that the self-monitoring approach can lead to an increase in the walking activity followed by improvement in the walking ability, which is substantially supportive of the theory that a higher dose of walking activity is necessary in improving walking ability in rehabilitation.

The most promising result was shown in the studies with stable COPD. The pathophysiology lies in deconditioning due to decreased activity and is considered to be a major cause of limb muscle dysfunction and subsequent decreased walking ability in COPD (39); however, there are no problems in the motor system of the gait. Therefore, the self-monitoring approach to motivate increasing activity might be a direct and effective solution for the walking ability in cases of COPD. Contrarily, neurological diseases such as stroke or Parkinson's disease have primary problems in the motor system of the gait, and thus there might be difficulty in obtaining the desired effect by simply motivating voluntary walking activity.

Another perspective is the indication according to the baseline physical function. Generally, pre-intervention walking ability or activity may play an important role in the effectiveness of walking interventions. The findings in patients with stable COPD in this review, in which a significant between-group difference was observed in the change from baseline, but not in the final value, suggest that populations with lower walking ability than their potential may benefit from this intervention to a larger extent. Moreover, in a study on chronic stroke (20), regression analysis and a sub-comparison involving participants with lower baseline values revealed that the self-monitoring approach was substantially more effective in participants with limited baseline walking activity and low baseline 6-min walking distance. Thus, the self-monitoring approach may be more effective for those with lower initial abilities or activities. Further studies are required to confirm the most appropriate indications for the self-monitoring approach based on the baseline walking ability or activity regardless of the type of disease.

Although patients with stroke are believed to experience benefits from increased walking activity to gain functional recovery and for secondary stroke prevention, this review revealed little evidence of the benefit of the self-monitoring approach for enhancing walking ability in stroke rehabilitation. However, the results were obtained from single studies, each from different stages. One study (21) recruited stroke participants within 35 days from onset and the other (20) recruited stroke participants whose onset was more than 6 months previously, which are categorized as the “early subacute” and “chronic” phases, respectively, according to the framework recommended by the Stroke Recovery and Rehabilitation Roundtable (40). Since the time post-stroke is extremely important in planning stroke rehabilitation, more studies in each of the phases would be necessary to determine the optimal use of activity trackers for patients with stroke.

Regarding the setting of the studies, only two (21, 28) recruited inpatients during rehabilitation. Inpatients have the potential to experience substantial gains in walking ability, although they may experience difficulties in increasing walking distance in closed and monotonous environments compared to the outpatients targeted in most of the included studies. Comparing the amount of walking (time or steps per day) in inpatients and outpatients are required to discuss this issue.

### Specific Means of Self-Monitoring

Most studies adopted the means of providing activity data and feedback directly from physiotherapists face-to-face. However, the provision of information using Internet applications was also performed in some studies. A slight advantage might exist for providing tailored advice or appraisal beneficial in encouraging walking activities from the rehabilitation staff, although this review obtained inadequate findings to draw any concrete conclusions. This should be evaluated in further studies considering the cost-effectiveness of the intervention.

Regarding setting goals, the self-monitoring approach with any trend in favor of intervention groups were showing specific goals to be achieved. Setting appropriate goals might be an important factor in performing the self-monitoring approach as part of rehabilitation (41).

### Limitations

As limitations, we described the studies with some concerns regarding the risk of bias in the results as well as the study with an absolutely low risk of bias. The synthesized key findings should thus be interpreted with caution when applied to clinical settings. However, as mentioned in the assessment of risk of bias, the most prevalent risk was due to the nature of the intervention, which aimed to manifest the amount of activity and considered to have fewer concerns than in usual medical interventions assessed by the Cochrane Risk of Bias tool. Finally, there was publication restriction due to the exclusive inclusion of English literature.

## Conclusions

This systematic review identified 11 studies on patients with eight different disease types that investigated the effectiveness of an additional self-monitoring approach using fitness trackers to improve walking ability. The findings indicated that there was little, if any, evidence of the effectiveness of the self-monitoring approach using fitness trackers in rehabilitation settings as a whole, whereas there was weak recommendation for patients with stable COPD implied in a quantitative synthesis. Nevertheless, the self-monitoring approach could be an alternative intervention for motivating rehabilitation patients when an appropriate population is selected, as this method using a consumer-based pedometer is relatively inexpensive and practical (42).

Despite the limitations listed above, this review provides insight into the impact of the self-monitoring approach in clinical rehabilitation settings. Moreover, considering the findings in this review, future research strategies are recommended to explore the best indications for this self-monitoring approach, including the types of diseases and baseline physical function or physical activities.

## Data Availability Statement

The raw data supporting the conclusions of this article will be made available by the authors, without undue reservation.

## Author Contributions

EO and YO determined the concept and design, performed study selection and data extraction, analyzed and interpreted the data, and edited the manuscript. KO participated in concept determination, data interpretation, manuscript editing, and critical revisions of the manuscript. IK participated in data interpretation and critical revisions of the manuscript. All authors contributed to the article and approved the submitted version.

## Conflict of Interest

The authors declare that the research was conducted in the absence of any commercial or financial relationships that could be construed as a potential conflict of interest.

## Publisher's Note

All claims expressed in this article are solely those of the authors and do not necessarily represent those of their affiliated organizations, or those of the publisher, the editors and the reviewers. Any product that may be evaluated in this article, or claim that may be made by its manufacturer, is not guaranteed or endorsed by the publisher.
