# Supplementary material for: Effectiveness of Self-Monitoring Approach Using Fitness Trackers to Improve Walking Ability in Rehabilitation Settings: A Systematic Review
Source: Front Rehabil Sci. 2021 Dec 2;2:752727. doi: 10.3389/fresc.2021.752727 (PMC9397729; doi:10.3389/fresc.2021.752727)
Supplement: Supplementary file 1 [file Data_Sheet_1.PDF]

Appendix A. Search strategies in electronic databases.

PubMed (MEDLINE)

#1

(fitness trackers[MeSH] OR “activity monitor”[TIAB] OR “pedometer”[TIAB] OR ”accelerometer”[TIAB] OR “actigraph”[TIAB]) AND clinical trial[ptyp]

#2

(motor activity[MeSH] OR physical activity[TIAB]) AND (rehabilitation[MeSH] OR physical therapy modalities[MeSH]) AND clinical trial[ptyp] AND "published last 10 years"[Filter]

#3

#1 AND #2

SPORTDiscus with Full Text

AB (“fitness tracker” OR “activity monitor” OR “pedometer” OR “accelerometer” OR “actigraph”) AND AB (motor activity OR physical activity) AND AB (rehabilitation OR physical therapy)

Cochrane Library

“fitness tracker” OR “activity monitor” OR “pedometer” OR “accelerometer” OR “actigraph” in Title Abstract Keyword AND "motor activity" OR "physical activity" in Title Abstract Keyword AND rehabilitation OR "physical therapy" in Title Abstract Keyword - with Cochrane Library publication date Between Nov 2009 and Nov 2019 (Word variations have been searched)

CINAHL

AB (“fitness tracker” OR “activity monitor” OR “pedometer” OR “accelerometer” OR “actigraph) AND AB (motor activity OR physical activity) AND AB (rehabilitation OR physical therapy)
