# Supplementary material for: Effectiveness of Self-Monitoring Approach Using Fitness Trackers to Improve Walking Ability in Rehabilitation Settings: A Systematic Review
Source: Front Rehabil Sci. 2021 Dec 2;2:752727. doi: 10.3389/fresc.2021.752727 (PMC9397729; doi:10.3389/fresc.2021.752727)
Supplement: Supplementary file 2 [file Data_Sheet_2.PDF]

Appendix B. Summary of the excluded studies.

Study	Reason for exclusion
Altenburg 2014[1]	no conventional rehabilitative intervention for control group
Arbillaga-Etxarri 2018[2]	no conventional rehabilitative intervention for control group
Bohm 2014[3]	not designed for investigating effectiveness of pedometer self-monitoring
Coleman 2017[4]	no conventional rehabilitative intervention for control group
Duscha 2018[5]	no conventional rehabilitative intervention for control group
Ellis 2018[6]	not designed for investigating effectiveness of pedometer self-monitoring
Focht 2014[7]	not designed for investigating effectiveness of pedometer self-monitoring
Frederix 2015[8]	not designed for investigating effectiveness of pedometer self-monitoring
Frensham 2018[9]	not designed for investigating effectiveness of pedometer self-monitoring<br>no conventional rehabilitative intervention for control group
Gardner 2011[10]	not designed for investigating effectiveness of pedometer self-monitoring
Hornikx 2015[11]	not designed for investigating effectiveness of pedometer self-monitoring<br>no conventional rehabilitative intervention for control group
Johnson 2016[12]	no conventional rehabilitative intervention for control group
Karstoft 2013[13]	not designed for investigating effectiveness of pedometer self-monitoring<br>no conventional rehabilitative intervention for control group
Ng 2010[14]	not designed for investigating effectiveness of pedometer self-monitoring
Pinto 2013[15]	no conventional rehabilitative intervention for control group
Rogers 2015[16]	no conventional rehabilitative intervention for control group
Roos 2014[17]	no conventional rehabilitative intervention for control group

Tew 2015[18]	no conventional rehabilitative intervention for control group
Varas 2018[19]	no conventional rehabilitative intervention for control group
Vidoni 2016[20]	no conventional rehabilitative intervention for control group
Wan 2017[21]	no conventional rehabilitative intervention for control group
Widyastuti 2018[22]	not designed for investigating effectiveness of pedometer self-monitoring
Mansfield 2015[23]	not designed for investigating effectiveness of pedometer self-monitoring

References to studies excluded from this review

1. Altenburg WA, ten Hacken NH, Bossenbroek L, Kerstjens HA, de Greef MH, Wempe JB. Short- and long-term effects of a physical activity counselling programme in COPD: a randomized controlled trial. Respir Med. (2015) 109:112-21.
2. Arbillaga-Etxarri A, Gimeno-Santos E, Barberan-Garcia A, et al. Long-term efficacy and effectiveness of a behavioural and community-based exercise intervention (Urban Training) to increase physical activity in patients with COPD: a randomised controlled trial. Eur Respir J. (2018) 52.
3. Bohm C, Stewart K, Onyskie-Marcus J, Esliger D, Kriellaars D, Rigatto C. Effects of intradialytic cycling compared with pedometry on physical function in chronic outpatient hemodialysis: a prospective randomized trial. Nephrol Dial Transplant. (2014) 29:1947-55.
4. Coleman KJ, Caparosa SL, Nichols JF, et al. Understanding the Capacity for Exercise in Post-Bariatric Patients. Obes Surg. (2017) 27:51-8.
5. Duscha BD, Piner LW, Patel MP, et al. Effects of a 12-Week mHealth Program on FunctionalCapacity and Physical Activity in Patients With PeripheralArtery Disease. Am J Cardiol. (2018) 122:879-84.
6. Ellis TD, Cavanaugh JT, DeAngelis T, et al. Comparative Effectiveness of mHealth-Supported Exercise Compared With Exercise Alone for People With Parkinson Disease: Randomized Controlled Pilot Study. Phys ther. (2019) 99:203-16.
7. Focht BC, Garver MJ, Devor ST, et al. Group-mediated physical activity promotion and mobility in sedentary patients with knee osteoarthritis: results from the IMPACT-pilot trial. J Rheumatol. (2014) 41:2068-77.
8. Frederix I, Hansen D, Coninx K, et al. Medium-Term Effectiveness of a Comprehensive Internet-Based and Patient-Specific Telerehabilitation Program With Text Messaging Support for

Cardiac Patients: Randomized Controlled Trial. J Med Internet Res. (2015) 17:e185.
9. Frensham LJ, Parfitt G, Dollman J. Effect of a 12-Week Online Walking Intervention on Health and Quality of Life in Cancer Survivors: A Quasi-Randomized Controlled Trial. Int J Environ Res Public Health. (2018) 15.
10. Gardner AW, Parker DE, Montgomery PS, Scott KJ, Blevins SM. Efficacy of quantified home-based exercise and supervised exercise in patients with intermittent claudication: a randomized controlled trial. Circulation. (2011) 123:491-8.
11. Hornikx M, Demeyer H, Camillo CA, Janssens W, Troosters T. The effects of a physical activity counseling program after an exacerbation in patients with Chronic Obstructive Pulmonary Disease: a randomized controlled pilot study. BMC Pulm Med. (2015) 15:136.
12. Johnson ST, Lubans DR, Mladenovic AB, Plotnikoff RC, Karunamuni N, Johnson JA. Testing social-cognitive mediators for objective estimates of physical activity from the Healthy Eating and Active Living for Diabetes in Primary Care Networks (HEALD-PCN) study. Psychol Health Med. (2016) 21:945-53.
13. Karstoft K, Winding K, Knudsen SH, et al. The effects of free-living interval-walking training on glycemic control, body composition, and physical fitness in type 2 diabetic patients: a randomized, controlled trial. Diabetes Care. (2013) 36:228-36.
14. Ng NT, Heesch KC, Brown WJ. Efficacy of a progressive walking program and glucosamine sulphate supplementation on osteoarthritic symptoms of the hip and knee: a feasibility trial. Arthritis Res Ther. (2010)12:R25.
15. Pinto BM, Papandonatos GD, Goldstein MG, Marcus BH, Farrell N. Home-based physical activity intervention for colorectal cancer survivors. Psychooncology. (2013) 22:54-64.
16. Rogers LQ, Courneya KS, Anton PM, et al. Effects of the BEAT Cancer physical activity behavior change intervention on physical activity, aerobic fitness, and quality of life in breast cancer survivors: a multicenter randomized controlled trial. Breast Cancer Res Treat. (2015) 149:109-19.
17. Roos R, Myezwa H, van Aswegen H, Musenge E. Effects of an education and home-based pedometer walking program on ischemic heart disease risk factors in people infected with HIV: a randomized trial. J Acquir Immune Defic Syndr. (2014) 67:268-76.
18. Tew GA, Humphreys L, Crank H, et al. The development and pilot randomised controlled trial of a group education programme for promoting walking in people with intermittent claudication. Vasc Med. (2015) 20:348-57.
19. Varas AB, Córdoba S, Rodríguez-Andonaegui I, Rueda MR, García-Juez S, Vilaró J. Effectiveness of a community-based exercise training programme to increase physical activity level in patients with chronic obstructive pulmonary disease: A randomized controlled trial. Physiother Res Int. (2018) 23:e1740.
20. Vidoni ED, Watts AS, Burns JM, et al. Feasibility of a Memory Clinic-Based Physical Activity Prescription Program. J Alzheimers Dis. (2016) 53:161-70.
21. Wan ES, Kantorowski A, Homsy D, et al. Promoting physical activity in COPD: Insights from

a randomized trial of a web-based intervention and pedometer use. Respir Med. (2017) 130:102-10.
22. Widyastuti K, Makhabah DN, Setijadi AR, Sutanto YS, Suradi, Ambrosino N. Benefits and costs of home pedometer assisted physical activity in patients with COPD. A preliminary randomized controlled trial. Pulmonology. (2018) 24:211-8.
23. Mansfield A, Wong JS, Bryce J, et al. Use of Accelerometer-Based Feedback of Walking Activity for Appraising Progress With Walking-Related Goals in Inpatient Stroke Rehabilitation: A Randomized Controlled Trial. Neurorehabil Neural Repair. (2015) 29:847-57.
