# Supplementary figures and images for: Effectiveness of Self-Monitoring Approach Using Fitness Trackers to Improve Walking Ability in Rehabilitation Settings: A Systematic Review
Source: Front Rehabil Sci. 2021 Dec 2;2:752727. doi: 10.3389/fresc.2021.752727 (PMC9397729; doi:10.3389/fresc.2021.752727)

**Appendix C.** Funnel plot for walking endurance post-intervention in various diseases.

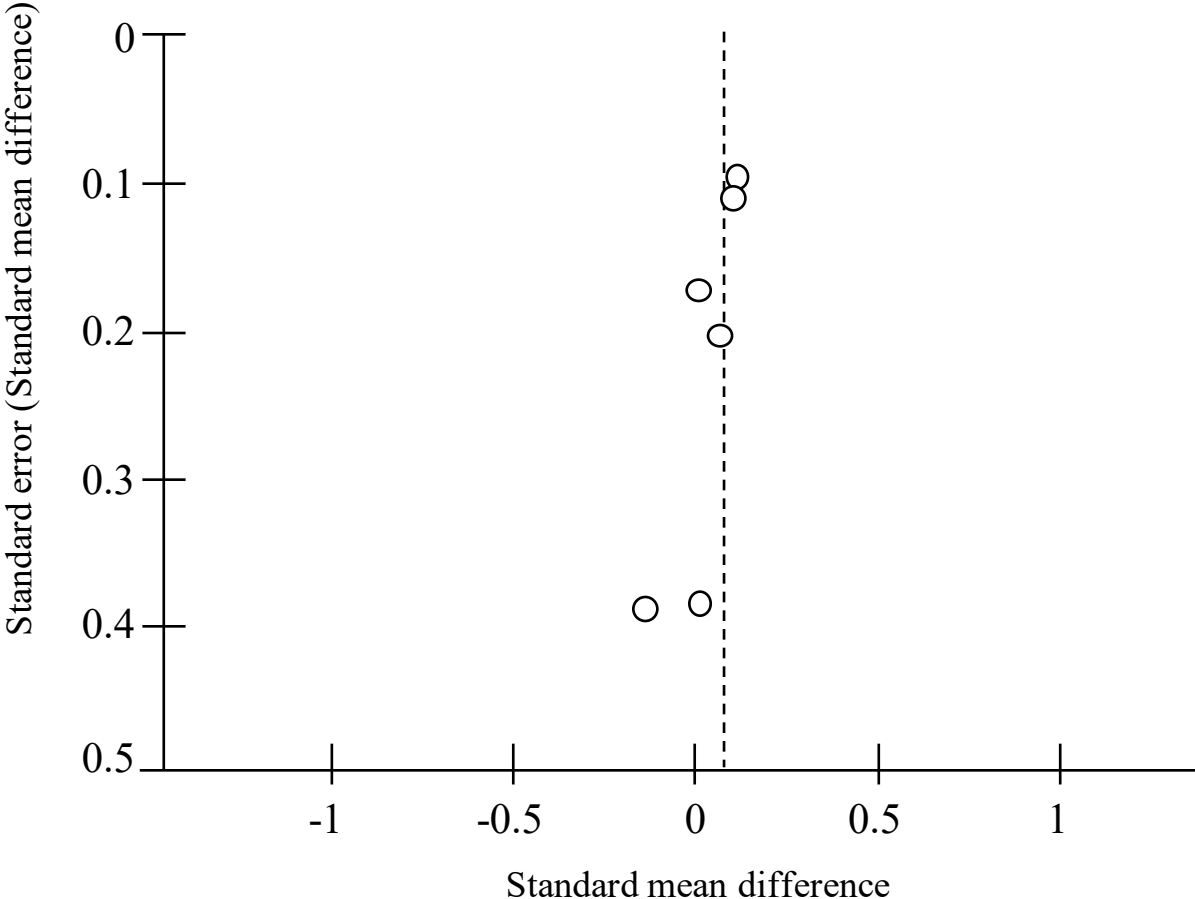

Supplement: Supplementary file 3 [file Data_Sheet_3.PDF]
